# Method for estimating pulsatile wall shear stress from one‐dimensional velocity waveforms

**DOI:** 10.14814/phy2.15628

**Published:** 2023-04-17

**Authors:** J. C. Muskat, C. F. Babbs, C. J. Goergen, V. L. Rayz

**Affiliations:** ^1^ Weldon School of Biomedical Engineering Purdue University West Lafayette Indiana USA; ^2^ Mechanical Engineering Purdue University West Lafayette Indiana USA

**Keywords:** flow‐mediated dilation, reduced‐order model, shear rate, velocity profile, Womersley solution

## Abstract

Wall shear stress (WSS)—a key regulator of endothelial function—is commonly estimated in vivo using simplified mathematical models based on Poiseuille's flow, assuming a quasi‐steady parabolic velocity distribution, despite evidence that more rapidly time‐varying, pulsatile blood flow during each cardiac cycle modulates flow‐mediated dilation (FMD) in large arteries of healthy subjects. More exact and accurate models based on the well‐established Womersley solution for rapidly changing blood flow have not been adopted clinically, potentially because the Womersley solution relies on the local pressure gradient, which is difficult to measure non‐invasively. We have developed an open‐source method for automatic reconstruction of unsteady, Womersley‐derived velocity profiles, and WSS in conduit arteries. The proposed method (available online at https://doi.org/10.5281/zenodo.7576408) requires only the time‐averaged diameter of the vessel and time‐varying velocity data available from non‐invasive imaging such as Doppler ultrasound. Validation of the method with subject‐specific computational fluid dynamics and application to synthetic velocity waveforms in the common carotid, brachial, and femoral arteries reveals that the Poiseuille solution underestimates peak WSS 38.5%–55.1% during the acceleration and deceleration phases of systole and underestimates or neglects retrograde WSS. Following evidence that oscillatory shear significantly augments vasodilator production, it is plausible that mischaracterization of the shear stimulus by assuming parabolic flow leads to systematic underestimates of important biological effects of time‐varying blood velocity in conduit arteries.

**New & noteworthy**

We developed an open‐source method for automatic reconstruction of pulsatile velocity profiles and wall shear stress from one‐dimensional velocity waveforms in conduit arteries obtained from clinically available time‐averaged diameter and time‐varying one‐dimensional velocity data, acquired in vivo with non‐invasive medical imaging. This new method of data analysis can provide substantially more accurate estimates of oscillatory shear stress affecting the arterial endothelium.

## 1 INTRODUCTION

Endothelial dysfunction precedes asymptomatic vascular remodeling and is involved in atherogenesis, leading to profound clinical manifestations of cardiovascular disease (Hadi et al., 2005). As pathological complications associated with atherosclerotic plaques, that is, myocardial infarction and stroke, remain leading causes of global mortality (GBD 2019 Diseases and Injuries Collaborators, 2020), the flow‐mediated dilation (FMD) test emerged as the non‐invasive standard for assessing endothelial function in vivo. However, concerns with accuracy, poor reproducibility, and high dependence on laboratory training (Thijssen et al., 2011) have limited translation of FMD to clinical use. Hence the exact clinical relevance and the best way to relate FMD to physiologic wall shear stress (WSS), which is equal to shear rate at the vessel wall multiplied by the blood viscosity, are still matters of debate (Thijssen et al., 2019).

Briefly, the FMD test produces a shear‐evoked, endothelium‐dependent dilatory response, typically in radial and ulnar arteries, during reactive hyperemia that occurs following complete occlusion of blood flow by blood pressure cuff around the upper arm. Early work by Celermajer et al. (Celermajer et al., 1992) assumed FMD to be caused by flow‐derived nitric oxide (NO) release from arterial endothelial cells, based upon available animal data. Contrary evidence of FMD despite NO blockade (Pyke et al., 2010; Stoner et al., 2012) has been attributed to differences in methodology and population groups (Thijssen et al., 2019), since a meta‐analysis (>8300 subjects) across 14 studies indicated placement of the occlusion cuff alters the shear stimulus and NO dependency of the resultant dilation (Green et al., 2011; Inaba et al., 2010). A more recent analysis by Green and colleagues (Green et al., 2014) demonstrated that FMD of conduit arteries in humans is largely (~70%) facilitated by NO.

Endothelium‐derived, NO‐mediated vasodilation is rate‐sensitive with dependency on both frequency and amplitude of the shear stimulus (Butler et al., 2000; Hutcheson & Griffith, 1991; Noris et al., 1995; Qiu & Tarbell, 2000) with evidence for oscillatory shear significantly upregulating NO production (Florian et al., 2003; Hillsley & Tarbell, 2002). Standardized FMD protocols (Parker et al., 2009; Thijssen et al., 2019) utilize single variable (i.e., non‐invasive ultrasonic peak or mean velocity) measurements to estimate WSS. The usual technique for estimating WSS assumes a quasi‐steady fully developed parabolic velocity profile based on Poiseuille flow. However, recent evidence indicates that higher frequency, unsteady fluctuations in shear rate, with unaltered mean shear, modulate FMD in healthy subjects (Holder et al., 2019; Stoner & McCully, 2012). We hypothesized that the Poiseuille assumption would lead to a biologically meaningful systematic underestimation of WSS and that more accurate results could be obtained by deriving time‐varying velocity profiles using Womersley theory.

To investigate how underlying assumptions influence reconstructed WSS in conduit arteries, we compared traditional Poiseuille reconstructions with a new, open‐source method for automatic reconstruction of time‐varying radial velocity profiles. The new method is based on the more physically exact and accurate Womersley solution for pulsatile blood flow, in which the radial velocity profile and subsequent WSS calculations are driven by time‐varying one‐dimensional (1D) centerline or mean velocity waveforms. Such waveforms can be acquired in vivo with non‐invasive medical imaging. The method was validated with subject‐specific computational fluid dynamics (CFD) simulations driven by in vivo pulsed‐wave Doppler (PWD) velocity recordings.

As representative test cases, we compared the time‐varying Womersley method to the quasi‐steady Poiseuille reconstruction using computer‐generated velocity waveforms for the right common carotid (RCC), brachial (RBRC), and femoral (RF) arteries at rest and during cardiovascular stress (i.e., fear and aerobic exercise). To check for accuracy, we compared the computer‐generated velocity profiles to experimental data from the brachial artery in a single test subject. Results show that estimating WSS based on the steady flow assumptions of Poiseuille's flow fails to capture physiologic levels of oscillatory shear stress.

## 2 METHODS

The authors declare that all supporting data are available within the article.

### 2.1 Simulated physiologic and hyperemic velocity waveforms

To compare WSS estimation methods for a variety of major arteries in the human body under varying conditions, we employed a previously developed reduced‐order model of the major systemic vessels within the trunk, limbs, and head at rest, during a fight‐or‐flight response (or fear, for short), and during moderate aerobic exercise (Muskat et al., 2021). The model consists of 83 systemic arteries with morphologies based upon extensive review of modern high‐resolution anatomical data sets with a focus on young, active humans (20–30 y.o.). In brief, the arterial network consists of three main parts: (1) a single ventricle pump, (2) transmission line segments, and (3) peripheral three‐element Windkessel boundaries connected to a lumped venous compartment to form a closed‐loop system. Rest state assumptions for a healthy young adult human included heart rate of 70 beats/min and maximum ventricular pressure of 110 mmHg. These were increased to 100 beats/min and 130 mmHg for fear and to 150 beats/min and 150 mmHg for exercise. We assumed that blood is an incompressible Newtonian fluid with density (*ρ*) of 1060 kg/m^3^ and viscosity (*μ*) of 4.5 mPa·s. The flow in all segments is assumed to be laminar.

**FIGURE 1:**
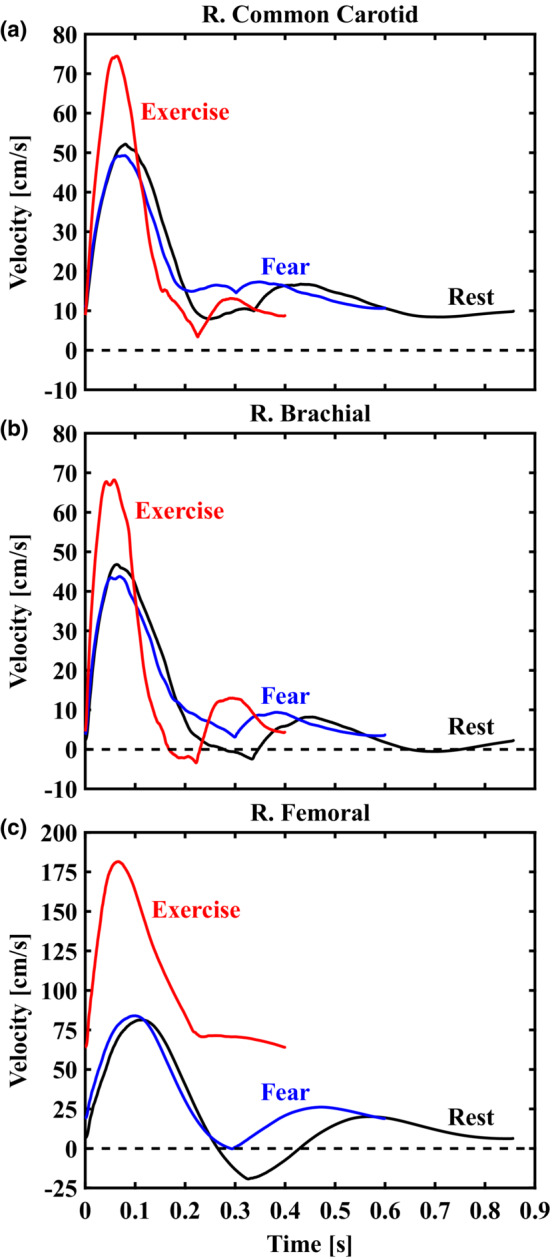
Representative 1D velocity waveforms (*U*_1D_(*t*)) output from the reduced‐order model presented in Muskat et al. (Muskat et al., 2021) for the right common carotid (a), brachial (b), and femoral (c) arteries. Computed waveforms for rest (70 beats/min), fear (100 beats/min), and exercise (150 beats/min) states are displayed in black, blue, and red curves, respectively. These idealized data provided inputs for our initial tests of the Poiseuille‐ and Womersley‐derived estimates of wall shear stress.

Input 1D velocity waveforms (*U*_1D_(*t*)), as shown in Figure 1, were obtained from the middle segment of each artery for all cardiovascular states. In addition, we obtained pressure waveforms from the transmission line model at the same locations and replicated the pressure‐corrected Womersley solution proposed by Azer and Peskin (Azer & Peskin, 2007) to test and evaluate the improved accuracy of our velocity‐driven solution. Further details on the numerical method and models of cardiovascular stress are described in Muskat et al. (Muskat et al., 2021). Access to the MATLAB R2017b scripts and supplementary files containing boundary conditions and morphological data are available at https://doi.org/10.5281/zenodo.4630326.

### 2.2 Poiseuille reconstruction of steady and quasi‐steady WSS

Poiseuille's flow accounts for the steady, fully developed motion of flowing blood (i.e., the time‐averaged mean flow component). The following equations indicate a separate problem for each axial location where *U*_1D_(*t*) is acquired. Assuming a parabolic radial velocity profile, the steady component of blood flow is expressed by Poiseuille's flow following Equation (1):

(1) $$U_{\mathrm{prb}}\left(r\right)=2\bar{U}\left[1-{\left(\frac{r}{R}\right)}^{2}\right]$$

where $\bar{U}$ is time‐averaged mean velocity of *U*_1D_(*t*) and *r* is the radial location ranging from 0 to the vessel radius *R* (in practice, the time‐averaged mean radius from the reduced‐order model is used for *R*). Steady flow rate ($\bar{Q}$) is obtained by integrating the velocity field on the cross‐section:

(2) $$\bar{Q}=\int_{0}^{R}2\pi \bar{U}\mathrm{rdr}=\bar{U}\pi R^{2}$$

Furthermore, the steady component of WSS (${\bar{\tau }}_{w}$) is:

(3) $${\bar{\tau }}_{w}=\frac{4\mu \bar{U}}{R}$$

as commonly employed in studies of flow‐mediated dilation (Parker et al., 2009). For arterial flow with an assumed parabolic velocity distribution, Equations (GBD 2019 Diseases and Injuries Collaborators, 2020; Hadi et al., 2005; Thijssen et al., 2011) may be modified to consider instantaneous velocity (i.e., direct input of *U*_1D_(*t*) rather than $\bar{U}$), thereby producing terms with time dependency: $\bar{U}$(*r*, *t*), $\bar{Q}$(*t*), and ${\bar{\tau }}_{w}$(*t*). This “quasi‐steady” approximation assumes frequency content is negligible and refers to reconstruction of transient flow profiles with respect to Poiseuille's flow (Leguy et al., 2009).

### 2.3 Womersley reconstruction of unsteady WSS

Womersley theory provides analytical solutions for reconstructing the radial velocity profile (*U*(*r*, *t*)), flow rate (*Q*(*t*)), and WSS (*τ*_*w*_(*t*)) at any arterial segment based on previous work (Leguy et al., 2009; Leow & Tang, 2018; Wei et al., 2019) for laminar pulsatile flow when the axial pressure gradient is known (Womersley, 1955). Unlike the original Womersley derivation, our method is driven by 1D velocity waveforms (*U*_1D_(*t*)) as depicted in Figure 1. The publicly available code (https://doi.org/10.5281/zenodo.7576408) has been simplified to require only time‐averaged vessel diameter and time‐varying 1D velocity data from the user. The Fourier and Bessel functions representing the velocity profile below are automatically computed from input data, allowing users to interact minimally with the mathematical details of the method. In particular, the Womersley velocity profile is expressed by Equations (4) and (5):

(4) $$U_{1D}\left(t\right)=\sum_{n=0}^{N}B_{n}e^{\text{inωt}}$$

(5) $$U\left(r,t\right)=2B_{0}\left[1-{\left(\frac{r}{R}\right)}^{2}\right]+\sum_{n=1}^{N}B_{n}\left[\frac{1-\frac{J_{0}\left(\Lambda_{n}\frac{r}{R}\right)}{J_{0}\left(\Lambda_{n}\right)}}{1-\frac{2J_{1}\left(\Lambda_{n}\right)}{\Lambda_{n}J_{0}\left(\Lambda_{n}\right)}}\right]e^{\text{inωt}}$$

with *Λ*_*n*_ = *αn* ^1/2^ *i* ^3/2^ used for simplification where the dimensionless Womersley number, *α* = *R*(*ωρ*/*μ*)^1/2^, defines the shape of the radial velocity profile through relation of vessel radius, pulsatile flow frequency, and viscous forces. Here *ω* is the angular frequency of the cardiac pulse, *t* is time, *n* is the natural numbers, and *i* is the imaginary number. *B*_*n*_ is Fourier coefficients that decompose the time‐varying *U*_1D_(*t*) wave. *J*_0_ and *J*_1_ denote Bessel functions of the first kind of order 0 and 1, respectively. The first term in Equation (5) represents the quasi‐steady state (Poiseuille) component or average velocity. The second term in Equation (5) represents the time‐varying component. Neglecting the steady component for now, time‐varying flow rate is obtained by integrating the velocity field over the vessel's cross‐section:

(6) $$Q\left(t\right)=\int_{0}^{R}2\mathrm{\pi U}\left(r,t\right)rdr=\text{Real}\left\{\pi R^{2}\sum_{n=1}^{N}B_{n}\left[\frac{1-\frac{2J_{1}\left(\Lambda_{n}\right)}{\Lambda_{n}J_{0}\left(\Lambda_{n}\right)}}{1-\frac{2J_{1}\left(\Lambda_{n}\right)}{\Lambda_{n}J_{0}\left(\Lambda_{n}\right)}}\right]e^{\text{inωt}}\right\}$$

Also, neglecting the steady component for now, the time‐varying component of WSS is obtained by multiplying time‐varying shear rate by viscosity:

(7) $$\tau_{w}\left(t\right)=\mu \left.\frac{dU\left(r,t\right)}{dr}\right|\begin{array}{c} \;\\ \;\\ r=R \end{array}=\text{Real}\left\{\mu \sum_{n=1}^{N}B_{n}\left[\frac{\frac{\Lambda_{n}J_{1}\left(\Lambda_{n}\right)}{RJ_{0}\left(\Lambda_{n}\right)}}{1-\frac{2J_{1}\left(\Lambda_{n}\right)}{\Lambda_{n}J_{0}\left(\Lambda_{n}\right)}}\right]e^{\text{inωt}}\right\}$$

**FIGURE 2:**
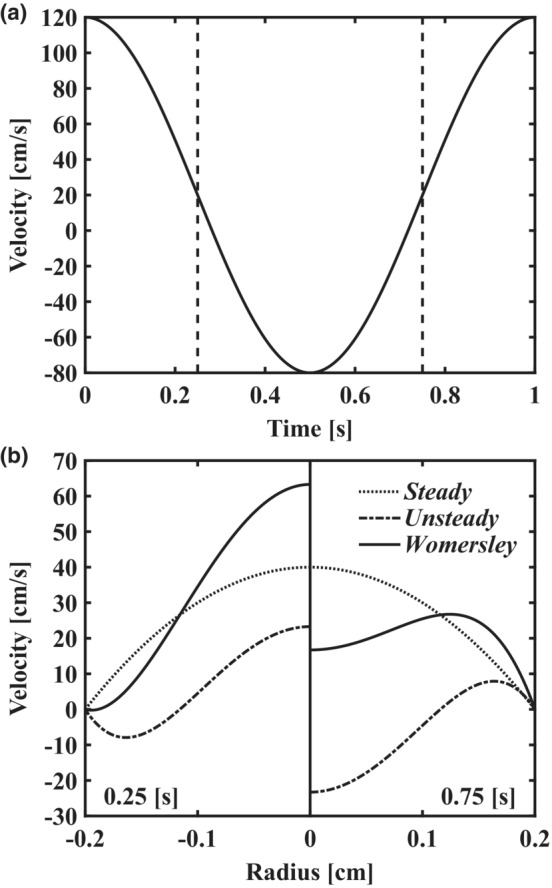
Demonstration of methods using an analytical solution for pulsatile flow in a straight pipe. (a) input velocity waveform as a cosine function with time‐averaged mean velocity of 20 cm/s and period of 1.0 s. (b) Womersley velocity profiles (solid lines) at 0.25 s (left subpanel) and 0.75 s (right subpanel) with steady (dotted lines) and unsteady (dash‐dotted lines) components displayed separately. Steady and unsteady components are combined to produce the Womersley solution as illustrated in each subpanel. The steady component defined by Poiseuille's flow neglects the effects of acceleration and deceleration which are shown to modulate the radial velocity profile across the cardiac cycle.

Equations (6) and (7) are solved for the first 20 harmonics and combined with their respective steady (*n* = 0th harmonic) components as defined in Equations (2) and (3) to complete the Womersley summation. The summation of steady and unsteady components is demonstrated in Figure 2 as applied to an input sinusoidal test waveform. Note the “steady” component is matched for each subpanel, demonstrating how Poiseuille's flow does not account for temporal variations in velocity (i.e., the influence of acceleration and deceleration).

### 2.4 Defining the oscillatory shear index

The oscillatory shear index (OSI) characterizes how the WSS vector deflects from the predominant direction of flowing blood across the cardiac cycle (Soulis et al., n.d.). For example, OSI may be appreciated as the fraction of each cardiac cycle where the endothelium experiences retrograde WSS (i.e., shear stress directed proximally). Thus, OSI is calculated as:

(8) $$\mathrm{OSI}\left(x\right)=0.5\left[1-\frac{\left|\int_{0}^{T}\tau_{w}\left(t\right)dt\right|}{\int_{0}^{T}\left|\tau_{w}\left(t\right)dt\right|}\right]$$

where *T* is the period of the cardiac cycle. The OSI ranges from 0, indicating non‐reversing WSS, to a maximum value of 0.5.

### 2.5 Validation with subject‐specific medical imaging and CFD

The following section provides information on magnetic resonance angiography (MRA) and ultrasound imaging studies used to validate the WSS predictions of the velocity‐driven Womersley solution with solutions of three‐dimensional CFD WSS.

#### 2.5.1 MR and ultrasound acquisition

Time‐of‐flight MRA in the right arm of a healthy male subject (27 y.o.) was obtained on a 3T MAGNETOM Prisma scanner (Siemens) at the Purdue MRI Facility, Purdue University. MRA scan parameters were repetition time (TR), 27 ms; echo time (TE), 3.58 ms; flip angle, 18°; pixel bandwidth, 185; and spatial resolution, 0.38 × 0.38 × 0.44 mm. High‐resolution color and PWD velocity recordings were obtained in the same subject with a Vevo 3100 imaging platform and a 15–30 MHz frequency linear array transducer (MX250, FUJIFILM VisualSonics Inc.). Collected PWD waveforms were extracted with a custom detection script (MATLAB R2017b, MathWorks) (Phillips et al., 2017). All study procedures were approved by the Purdue University Institutional Review Board.

#### 2.5.2 Computational methods

**FIGURE 3:**
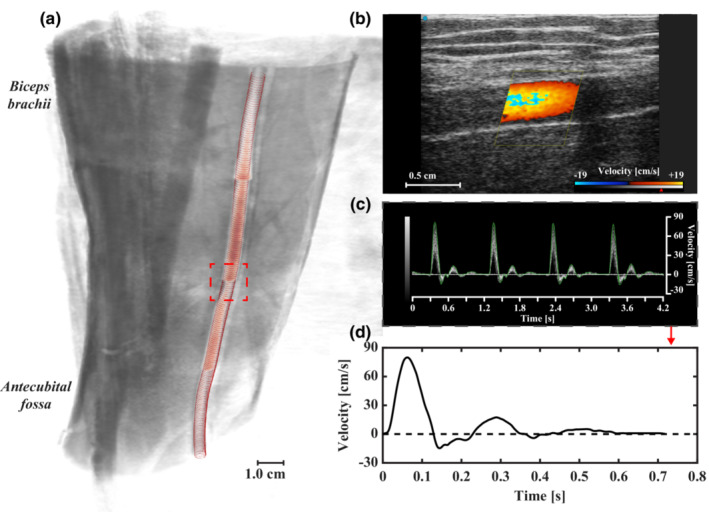
Morphological and hemodynamic data acquired for comparison to computational fluid dynamics (CFD). (a) Volume rendering of brachial artery time‐of‐flight magnetic resonance angiography (MRA) in healthy adult male subject. MRA localized to 18 × 20 × 15 cm of upper arm proximal to the antecubital fossa. Brachial artery segmentation overlaid in red. Region of interest (dashed lines) where (b) color and (c) pulsed‐wave Doppler were acquired. (d) One‐dimensional time‐varying velocity waveform extracted from ultrasound data at the midsection of the region of interest. Physiologic brachial artery waveform indicated by triphasic blood flow.

Open‐source software package SimVascular (Updegrove et al., 2017) was used for segmentation of MRA data as shown in Figure 3a. An unstructured tetrahedral mesh with a refined boundary layer was generated in HyperMesh 2021 (Altair Engineering Inc.) with a target edge length of 300 μm, leading to a total of 516,000 elements. Mesh independence was verified as less than 5% deviation of maximum WSS predicted on a refined mesh with a 100 μm edge length. Meshes were transferred to the finite‐volume package, ANSYS Fluent v18.1 (Ansys Inc.) to execute CFD simulations with a temporal resolution of 0.5 ms. Arterial flow was assumed to be laminar with blood modeled as an incompressible Newtonian fluid with density (*ρ*) of 1060 kg/m^3^ and viscosity (*μ*) of 4.5 mPa·s. The 1D time‐varying velocity waveform extracted from PWD (Figure 3d) was prescribed at the inlet to drive the simulation, replicated for four cardiac cycles to ensure numeric stability. We quantified time‐varying WSS for the final cardiac cycle approximately 8 cm proximal to the antecubital fossa (see Figure 3) in ParaView v5.10.0 (Kitware Inc., Clifton Park, NY) (Ahrens et al., 2005) to be used as ground truth for brachial artery WSS distributions.

## 3 RESULTS

### 3.1 Validation of pulsatile wall shear stress predictions

**FIGURE 4:**
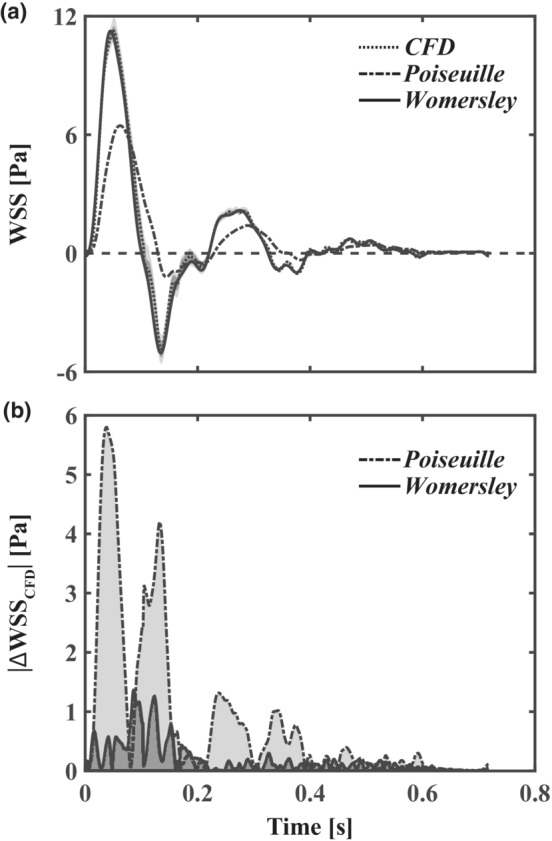
Validation of Womersley theory with computational fluid dynamics (CFD). (a) time‐varying wall shear stress (WSS) distributions with respect to subject‐specific CFD (median, dotted line; interquartile Q1–Q3 range, dark gray; and total range, light gray), Poiseuille's flow (dash‐dotted lines), and Womersley theory (solid lines). (b) Absolute WSS difference from ground truth CFD simulations for Womersley and Poiseuille solutions. The deviation of the Poiseuille solution from CFD is increased during periods of high‐velocity acceleration.

Here the Poiseuille and Womersley flow models are first compared using experimentally measured velocity from the brachial artery in a single subject, followed by CFD reconstructions of complete velocity profiles. The results in Figure 4 demonstrate the fundamental assumptions associated with Womersley theory (e.g., pulsatile axial flow in a long cylindrical tube) are valid for non‐bifurcating segments of large conduit arteries in humans.

#### 3.1.1 Subject‐specific CFD

WSS varied circumferentially at the cross‐section corresponding to the level of PWD measurement (i.e., 8 cm proximal to the antecubital fossa highlighted in red in Figure 3a). We illustrate this variance in Figure 4a by representing first and third quartiles, and total WSS range, in shaded gray. Results indicate the symmetrical pipe assumption of the Womersley solution is reasonable for the brachial artery as the 1D velocity‐driven method closely represents the CFD and interquartile Q1–Q3 WSS varied less than 5% circumferentially during systole. Importantly, pulsatile WSS estimated with Womersley theory was consistently within the range of WSS calculated with CFD. Relative to CFD, differences in Womersley peak and time‐averaged median WSS (0.03% and 19.9%, respectively) were lower than Poiseuille estimates (42.6% and 115.3%, respectively). Figure 4b illustrates that the error in WSS estimates is higher during the acceleration and deceleration phases of the centerline velocity. In particular, the Poiseuille solution fails to accurately represent WSS during periods of high‐velocity acceleration.

#### 3.1.2 Pressure‐ vs. velocity‐driven Womersley solution

We evaluated performance of the velocity‐driven Womersley model against the original pressure‐driven solution (Womersley, 1955) using the transmission line model detailed in (Muskat et al., 2021) to acquire instantaneous flow, diameter, and axial pressure gradients for each arterial segment. Analytical flow rates and transient velocity profiles for each Womersley solution demonstrated that the peak flow rate for the velocity‐driven solution was within 8.5% of the input waveform and within 13.5% for the pressure‐driven solution (supplemental data available online at https://doi.org/10.5281/zenodo.7576408). These discrepancies can be attributed to the pressure gradient obtained from the solution of one‐dimensional equations of an elastic tube, indicating nontrivial dependence on axial position and diameter as in (Azer & Peskin, 2007).

### 3.2 Velocity profile reconstruction

To expand the pool of test data to a larger set of arteries, we applied the Poiseuille and Womersley reconstructions to time‐averaged mean velocity waveforms from our previously published reduced‐order model of the normal human arterial tree (Muskat et al., 2021). Using these simulated velocity datasets to represent ultrasound velocity measurements, we calculated full radial velocity profiles for the carotid, brachial, and femoral arteries at rest and during cardiovascular stress (i.e., fear and aerobic exercise) using both Poiseuille and Womersley flow models. Our previously developed models (Muskat et al., 2021) account for vascular compliance and heart rate changes associated with acute cardiovascular stress. Reynolds numbers (Re) indicate laminar flow for all baseline rest (RCC, Re = 797; RBRC, Re = 708; RF, Re = 1393) and fear cases (RCC, 769; RBRC, 677; RF, 1475). The only case where a transitional flow may occur was the femoral artery during exercise (RCC, 1150; RBRC, 1044; RF, 3141). Womersley numbers increased from the baseline rest case (RCC, *α* = 4.3; RBRC, *α* = 4.2; RF, *α* = 4.8) during fear (RCC, 5.2; RBRC, 5.2; RF, 5.9) and aerobic exercise (RCC, 6.3; RBRC, 6.2; RF, 7.1). These values indicate that the velocity fields in these larger arteries are defined by the unsteadiness of blood flow rather than viscous forces. When the Womersley number is close to 1, the velocity profile is closely approximated by Poiseuille's flow. However, for larger Womersley numbers, pulse frequency and phase lag between flow and pressure waveforms dictate the shape of the velocity profile and subsequent WSS.

**FIGURE 5:**
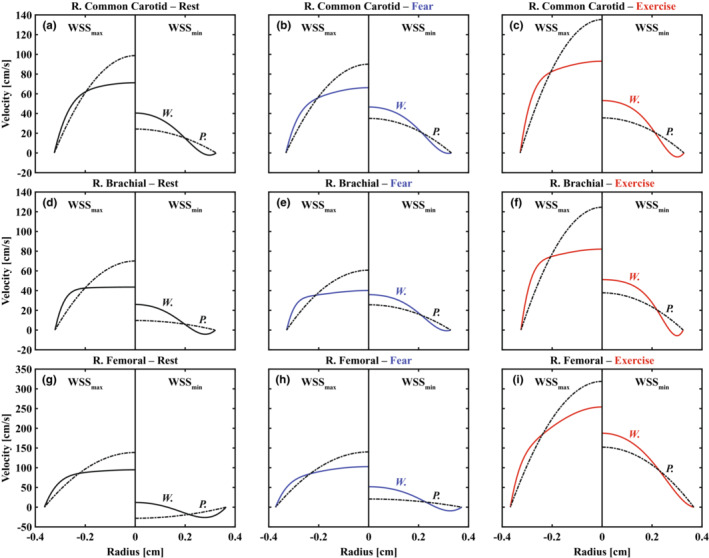
Reconstruction of transient radial velocity profiles using Poiseuille (P., dash‐dotted lines) and Womersley (W., solid lines) solutions for right common carotid (a–c), brachial (d–f), and femoral (g–i) arteries during rest (black), fear (blue), and exercise (red) states. Representative velocity profiles are shown for both methods at maximum (WSS_max_, left subpanels) and minimum (WSS_min_, right subpanels) WSS as determined via Womersley theory; therefore, the velocity fields shown here illustrate instantaneous variation between methods at matching time points. Negative slopes at the vessel wall in right subpanels mirror physiologic levels of oscillatory shear stress.

Figure 5 compares instantaneous velocity profiles calculated with Poiseuille and Womersley solutions at maximum (left subpanels) and minimum (right subpanels) WSS as determined with Womersley theory. Supplemental Videos detail transient velocity profiles throughout cardiac cycles at each location and are available online (https://doi.org/10.5281/zenodo.7576408). The Womersley profiles for the RF artery are similar for rest and fear states; however, during moderate aerobic exercise, significant increase in forward blood flow to the skeletal muscle prevented retrograde shear. In contrast, the flow reversal for the Poiseuille velocity model is possible only when mean velocity is reversed (see Figure 1). Further, in comparison to the Womersley solution, centerline velocity was consistently over‐ and underestimated during the acceleration and deceleration phases of systole, respectively.

### 3.3 Comparing estimates of wall shear stress

**FIGURE 6:**
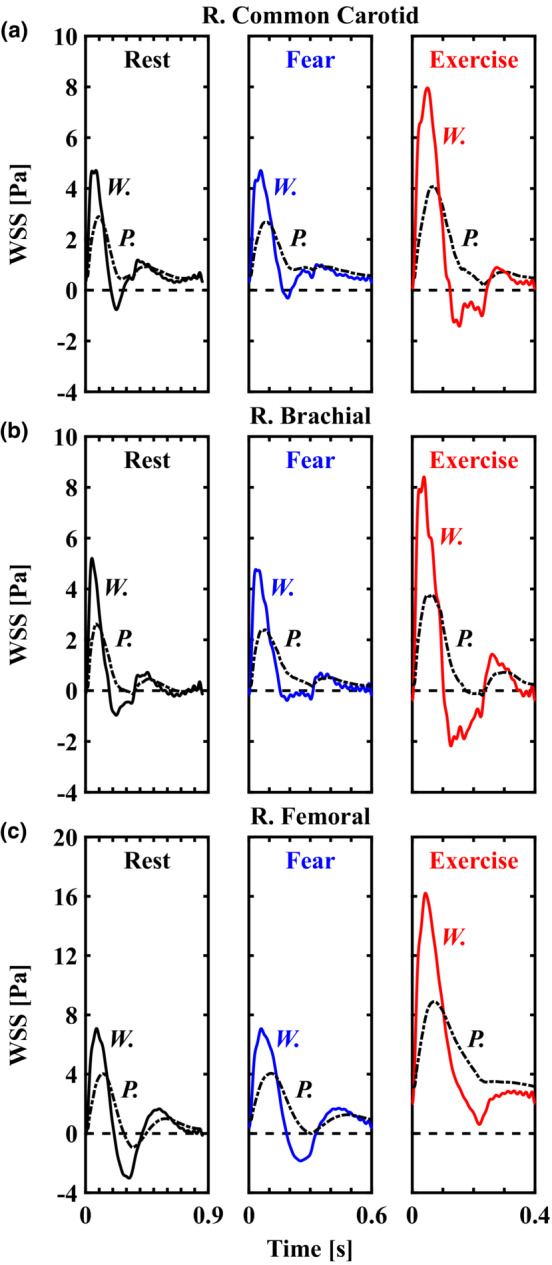
The effect of underlying flow assumptions on transient wall shear stress (WSS) in the major conduit arteries. WSS waveforms based on Poiseuille's flow (P., dash‐dotted lines) and Womersley theory (W., solid lines) for right common carotid (a), brachial (b), and femoral (c) arteries. Solutions for rest, fear, and exercise are displayed in black, blue, and red lines, respectively. Relative to the Womersley solution, Poiseuille's flow underestimated systolic WSS, overestimated diastolic WSS, and failed to capture negative values of WSS during the systolic deceleration phase. Differences are attributed to the unsteady component or rate of change in shear at the onset of flow being neglected by Poiseuille's flow.

**FIGURE 7:**
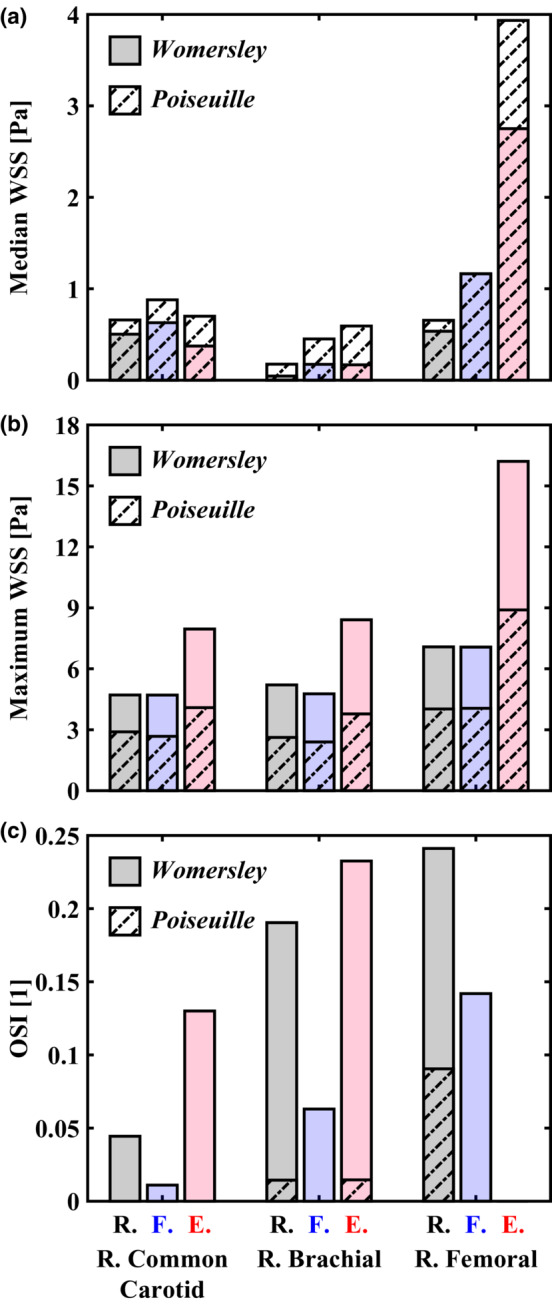
Hemodynamic metrics were evaluated with Poiseuille (dash‐dotted bars) and Womersley (solid bars) solutions for cardiovascular states of rest (R., black), fear (F., blue), and exercise (E., red). (a) Poiseuille's flow overestimated time‐averaged median wall shear stress (WSS) in conduit arteries. Underestimation (0.1%) in the femoral artery during a fear response was negligible. (b) in contrast, Poiseuille's flow consistently underestimated maximum WSS. Largest differences between methods occurred in the brachial artery. (c) evaluation of the oscillatory shear index (OSI) revealed physiologic levels of oscillatory shear stress occurred in conduit arteries despite fully monophasic, antegrade flows.

We evaluated transient WSS waveforms (Figure 6) for each method. Results indicate that reconstruction using Poiseuille's flow led to (1) overestimation of time‐averaged median WSS (RCC, 31.0%–87.4%; RBRC, 159.5%–285.1%; RF, −0.1% to 43.0%), (2) consistent underestimation of maximum WSS (RCC, 38.5%–48.6%; RBRC, 49.6%–55.1%; RF, 42.6%–45.1%), and (3) lack of an oscillatory shear component captured by the Womersley solution (see Figure 7). Differences in Poiseuille‐ and Womersley‐derived WSS were smallest in the femoral artery as larger time‐averaged mean velocity (i.e., greater weighting of the steady flow component) improved agreement between methods. Only the Womersley solution was capable of capturing reversal of the WSS direction relative to the antegrade flow. This aspect is important because input velocity waveforms for the RCC (see Figure 1) are monophasic and, following clinical guidelines of assuming parabolic flow, would otherwise indicate non‐reversing WSS. Capturing flow reversal is especially important in calculations of OSI, as shown in Figure 7c. Therefore, the predicted differences in OSI are attributed to the unsteady component or rate of change in shear during the acceleration and deceleration phases of systole. It follows that WSS waveforms shown in Figure 6 revealed largest deviation between methods during systole.

## 4 DISCUSSION

Recent evidence indicating that fluctuations in cyclic shear stress, with unaltered mean shear stress, augment FMD in healthy subjects has highlighted the need to further characterize the effect of transient shear stress patterns (Holder et al., 2019; Stoner & McCully, 2012). Our goal in this study was to clarify how fundamental assumptions regarding the physics of flow influence these patterns in conduit arteries. Calculation of the shear stimulus via single (i.e., peak velocity) or time‐averaged (i.e., mean velocity) variables neglect physiologic oscillatory shear stress (Figure 7c). The presented example cases reveal that assumptions of quasi‐steady flow with parabolic velocity distribution in large conduit arteries, frequently utilized in clinical studies, are not correct (Figure 7). These results highlight the need for an open‐source method based on a 1D time‐varying velocity measurement for automatic reconstruction of radial velocity profiles based on the Womersley solution for pulsatile flow.

Our findings agree with previous work indicating that Poiseuille‐based calculations underestimate amplitude of the pulsatile shear stimulus in comparison to Womersley theory (Mynard et al., 2013; Schwarz et al., 2015). For example, Gurovich and Braith found antegrade and retrograde shear rates are underestimated, each by over 50%, under resting conditions and moderate levels of aerobic exercise (40–70% of maximal oxygen consumption) in the brachial and femoral arteries using Poiseuille's flow (Gurovich & Braith, 2012). In each study, the authors concluded that the Womersley solution provided a better description of blood flow patterns.

Transient flow patterns are known to modulate endothelial production of vasodilators. For example, Hillsley and Tarbell identified a 2.9‐ and 2.6‐fold increase in NO production above non‐sheared controls in response to 1.0 and 2.0 ± 1.0 Pa, respectively; moreover, the addition of an oscillatory shear component with unaltered mean shear (i.e., 1.0 ± 1.5 Pa) stimulated a 14‐fold increase in NO synthesis (Hillsley & Tarbell, 2002). Since the FMD response of the brachial artery is facilitated by NO (Green et al., 2014) and NO‐mediated vasodilation is modulated by frequency and amplitude of the shear stimulus (Butler et al., 2000; Hutcheson & Griffith, 1991; Noris et al., 1995; Qiu & Tarbell, 2000), it follows that the unsteady, oscillatory component of shear stress is coupled to vasodilation and is thus critical to account for in vivo.

Some groups have attempted to characterize the effect of retrograde shear, but the results are conflicting. Green et al. demonstrated a dose‐dependent increase in brachial artery retrograde blood flow during cycling (Green, Cheetham, Reed, et al., 2002) and later confirmed a local increase in NO activity in the resting forearm (Green, Cheetham, Mavaddat, et al., 2002), suggesting that bidirectional flow improves forearm vascular function during exercise. However, the same group of investigators reported that brachial artery FMD was acutely impaired after 30‐minute exposure to retrograde flow conditions (Thijssen et al., 2009). This contradiction may be explained by the existence of a “threshold” for physiologically beneficial retrograde shear, representing a potent stimulus regulating endothelial function (Schreuder et al., 2014). The threshold hypothesis is supported by evidence that external counterpulsation, which produces robust retrograde blood flow in the femoral artery, increases peripheral artery FMD by 30%–50% (Braith et al., 2010).

Current FMD normalization techniques (i.e., dividing FMD by area‐under‐the‐curve or peak shear rate) neglect the unsteady, pulsatile component of flow and potentially underestimate maximum WSS and OSI in large arteries (see Figure 7). As these time‐varying effects have been neglected by Poiseuille‐derived methods, an interesting point arises when considering that factors which influence FMD variability (e.g., hypertension, age, gender, and baseline diameter (Thijssen et al., 2019)) are known to modulate the (1) amplitude and contour of the systemic pressure waveform and (2) subsequent arterial velocity waveforms. Without accounting for the velocity changes over the cardiac cycle with Womersley theory, an identical velocity recording between two time points, cardiac cycles, or days would suggest an identical velocity profile; however, acceleration and deceleration will alter the shear stimulus, resulting in large deviation from Poiseuille's flow (Figure 2b). Considering the effects of velocity acceleration and shear pattern on NO production and FMD (Holder et al., 2019; Stoner & Sabatier, 2012), we recommend estimation of the pulsatile shear stimulus via the Womersley solution.

### 4.1 Limitations

The methods described here are limited to healthy arteries. Stenotic, asymmetric, or more tortuous vessels with secondary flows do not adhere to fundamental assumptions (i.e., pulsatile axial flow in a long cylindrical tube) associated with Womersley's solution. The analytical solutions for reconstructing the instantaneous radial velocity profile, flow rate, and WSS at any arterial segment detailed here are driven by 1D velocity waveforms as acquired in vivo with Doppler ultrasound (Mynard et al., 2013; Stoner & McCully, 2012). While only time‐averaged diameter of the vessel and time‐varying mean velocity are required for this analysis, the temporal resolution of the measurements is of concern as the Fourier summation indicated in Equation 4 decomposes physiological waves into harmonics that constitute the original function. Therefore, accuracy of the method may be enhanced by using high frame rate ultrasound capable of providing multiple frames per second (Leow & Tang, 2018). Potential limitations with the idealized anatomy, where only a straight and rigid tube is considered, and the fundamental assumption of laminar flow exist. However, subject‐specific brachial artery WSS comparisons (see Figure 4) indicate long, non‐bifurcating arterial WSS estimates are accurately represented with Womersley theory. Peak antegrade and retrograde velocities in the brachial and femoral arteries suggest a transition from laminar flow at rest to turbulent flow during moderate aerobic exercise (Gurovich & Braith, 2012) that would not be captured with either Poiseuille‐ or Womersley‐derived methods. In addition, transitional blood flow occurs during ischemia‐induced reactive hyperemia which may limit the accuracy of WSS estimation using standard FMD protocols (Stoner et al., 2011). Lastly, we have not verified that reproducibility concerns associated with FMD (Thijssen et al., 2019) are coupled to errors in WSS estimation as discussed in this work. Future studies may utilize our method to efficiently determine how time‐varying dynamics of blood flow relate to FMD variability.

## 5 CONCLUSIONS

Underlying assumptions influence estimates of WSS in large conduit arteries. The assumption of a quasi‐steady, parabolic velocity profile leads to systematic bias and an underestimation of pulsatile WSS. To support clinical adoption of more accurate pulsatile flow solutions, we developed an open‐source method for automatic reconstruction of velocity profiles in time and space, based on the well‐established Womersley solution for pulsatile blood flow. The publicly available code (https://doi.org/10.5281/zenodo.7576408) requires only time‐averaged diameter of the vessel and time‐varying 1D velocity data from the user—readily acquired in vivo with non‐invasive medical imaging such as Doppler ultrasound. The method was validated with subject‐specific CFD simulations driven by in vivo PWD velocity recordings. In addition, we applied the method to synthetic 1D velocity waveforms in conduit arteries at rest and during two states of cardiovascular stress (i.e., fear and aerobic exercise). Relative to Womersley approximations, Poiseuille's flow underestimated systolic WSS, overestimated diastolic WSS, and failed to capture negative values of WSS during the systolic deceleration phase. These results are consistent across all representative cases (i.e., common carotid, brachial, and femoral arteries). In comparison to the Womersley solution, Poiseuille‐based calculations underestimated range of the WSS stimulus at the onset and offset of flow (i.e., during the acceleration and deceleration phases of the cardiac cycle) by as much as 63.1% and underestimated or failed to capture near‐wall reversal of WSS. Since fluctuations in shear patterns modulate vasodilation in vivo, these findings suggest that intra‐ and inter‐subject variability associated with FMD may be better related to transient WSS and oscillatory shear stress calculated with Womersley theory, rather than similar values calculated with the parabolic flow assumption of Poiseuille's flow.

## AUTHOR CONTRIBUTIONS

J. C. Muskat, C. F. Babbs, and V. L. Rayz conceived and designed research; J. C. Muskat and C. F. Babbs performed experiments; J. C. Muskat analyzed data; J. C. Muskat, C. F. Babbs, C. J. Goergen, and V. L. Rayz interpreted results of experiments; J. C. Muskat, C. F. Babbs, C. J. Goergen, and V. L. Rayz prepared figures; J. C. Muskat drafted manuscript; J. C. Muskat, C. F. Babbs, C. J. Goergen, and V. L. Rayz edited and revised manuscript; J. C. Muskat, C. F. Babbs, C. J. Goergen, and V. L. Rayz approved final version of manuscript.

## CONFLICTS OF INTEREST STATEMENT

No conflicts of interest, financial or otherwise, are declared by the authors.

## Supporting information


Data S1
